# Neuroimaging Analysis of the Dopamine Basis for Apathetic Behaviors in an MPTP-Lesioned Primate Model

**DOI:** 10.1371/journal.pone.0132064

**Published:** 2015-07-02

**Authors:** LinLin Tian, Yuanxuan Xia, Hubert P. Flores, Meghan C. Campbell, Stephen M. Moerlein, Joel S. Perlmutter

**Affiliations:** 1 Department of Neurology, Washington University, St. Louis, Missouri, United States of America; 2 Department of Radiology, Washington University, St. Louis, Missouri, United States of America; 3 Department of Neurobiology, Washington University, St. Louis, Missouri, United States of America; 4 Program of Occupational Therapy, Washington University, St. Louis, Missouri, United States of America; 5 Program of Physical Therapy, Washington University, St. Louis, Missouri, United States of America; University of Cincinnati, UNITED STATES

## Abstract

Apathy commonly occurs in Parkinson disease (PD) patients; however, the role of dopamine in the pathophysiology of apathy remains elusive. We previously demonstrated that dopaminergic dysfunction within the ventral tegmental area (VTA)-nucleus accumbens (NAcc) pathway contributes to the manifestation of apathetic behaviors in monkeys treated with the selective dopaminergic neurotoxin 1-methyl-4-phenyl-1,2,3,6-tetrahydropyridine (MPTP). We now extend these studies to identify dopaminergic dysfunction in cortical regions that correlate with development of apathetic behaviors. Specifically, we measured the effects of MPTP on monkeys' willingness to attempt goal directed behaviors, which is distinct from their ability to perform tasks. A total of 16 monkeys had baseline magnetic resonance imaging (MRI) and positron emission tomography (PET), using 6-[^18^F]fluorodopa (FD), [^11^C]dihydrotetrabenazine (DTBZ), and 2β-[^11^C]carbomethoxy-3β-(4-fluorophenyl)tropane (CFT). The monkeys received unilateral infusion of different doses of MPTP (0 – 0.31mg/kg) to produce a wide range of severity of motor parkinsonism. Eight weeks after MPTP, PET scans were repeated and animals were euthanized. Apathetic behavior and motor impairments were assessed blindly both pre- and post-MPTP infusion. Apathy scores were compared to *in vitro* and *in vivo* dopaminergic measures. Apathy scores increased following MPTP and correlated with PET measures of dopaminergic terminals (DTBZ or CFT) in dorsal lateral prefrontal cortex (DLPFC), ventromedial prefrontal cortex (VMPFC), and insular cortex (IC). Among all the cortical regions assessed, forward step-wise regression analyses indicated that only stereologic cell counts in VTA, and not counts in the substantia nigra (SN), predict dopamine transporter changes in IC. Our findings suggest that dopaminergic dysfunction within the VTA–IC pathway plays a role in the manifestation of apathetic behaviors in MPTP-lesioned primates.

## Introduction

Apathy, conventionally defined as a lack of motivation, is one of the most debilitating behavioral manifestations of Parkinson disease (PD) [1]. Functional imaging studies have shown involvement of several brain regions, such as dorsal lateral prefrontal cortex (DLPFC), ventromedial prefrontal cortex (VMPFC), cingulate cortex, and insular cortex (IC) [2–6]. However, the underlying neural mechanisms of apathy in PD remain largely unknown.

We previously quantified the behavioral features of apathy in monkeys using a validated apathetic behavior rating scale. This scale separates apathetic behavior manifestations from MPTP-induced motor impairment [7]. We found that the severity of apathetic behaviors in monkeys given different doses of unilateral internal carotid MPTP correlate with postmortem stereologic measures of reduced dopaminergic neurons in the ventral tegmental area (VTA) and with positron emission tomography (PET) measured specific binding of membranous dopamine transporter (DAT) and vesicular monoamine transporter type 2 (VMAT2) radioligands in nucleus accumbens (NAcc), a subcortical region. Forward step-wise regression analyses indicated that neuropathological changes in the VTA-NAcc pathway predicated apathetic behavior better than motor impairment or pathologic changes in the nigrostriatal pathway.

In the current study, we investigated whether apathetic behaviors in this animal model correlate with PET measures of dopaminergic dysfunction in several suspected cortical regions, such as DLPFC, VMPFC, anterior cingulate cortex (ACC), posterior cingulate cortex (PCC) and IC. We hypothesized that apathetic behaviors correlate with dopaminergic dysfunction in these cortical regions. Methodological improvement permitted us to extend our former findings in subcortical regions to cortical regions. Behavioral and biochemical data from all but one of these animals have been published in previous papers [7,8].

## Materials and Methods

### Ethics Statement

This study was approved by the Institutional Animal Care and Use Committee (IACUC) of Washington University in St. Louis and conducted by the Nonhuman Primate Facility at the Washington University School of Medicine. The protocol number is 20110161 and was approved on 6/25/2013. All procedures were designed to conform to suggestions of the 2006 Weatherall Report with steps taken to ameliorate subject suffering in accordance with the requirements of the National Institutes of Health (NIH). In the event of mild to moderate pain or discomfort, a primate would be treated with Buprenex, 0.01–0.03 mg/kg for up to three days, per the discretion of the staff veterinarian. No animals were knowingly exposed to potential infection and humane endpoints were pre-defined and applied, if necessary, to reduce subject discomfort. The macaques were housed in appropriate animal facilities with 12-hour day and 12-hour night cycles. They were provided with food and water *ad libitum* and toys and movies as enrichment activities. All animals were housed in the same USDA-approved facility room in cages that met or exceeded the Weatherall Report’s requirements. The animals were housed for approximately 6 months before pre-MPTP behavioral measures were initiated. The monkeys were monitored daily by the researchers, animal care staff, and a veterinarian, to check their general health and welfare. All routine animal care procedures have been described in detail [9].

### Subjects

Sixteen male macaque monkeys (13 *Macaca fascicularis* and 3 *Macaca nemestrina*) between the ages of 3.5 and 6.5 years old (mean age = 5.3 ± 0.9 years) were studied; data from one *Macaca fascicularis* monkey was added to the 15 monkeys used in our previous studies [7,8]. Two *Macaca fascicularis* monkeys were used as controls and were injected only with saline. We previously demonstrated that *Macaca fascicularis* and *Macaca nemestrina* had similar response to the same dose of MPTP [8,10] and similar apathetic behavioral responses as well [7].

### General Protocol

Detailed descriptions of the methods have been published [7,9–12] and are briefly described here. Primates were trained in several behavioral and motor exercises for at least 8 weeks prior to MPTP [7,10]. Training and subsequent rating sessions were performed mid-morning (less distraction for the primates) 2–3 times each week. All sessions before and after MPTP were recorded on digital video for at least 5 minutes. The primates also had one baseline structural MRI scan with a magnetization-prepared rapid acquisition gradient echo (MP-RAGE) sequence on a Siemens Trio 3T scanner: repetition time = 2,400 milliseconds, echo time = 3.93 milliseconds, inversion time = 1,100 milliseconds, FOV = 154 millimeter, flip angle = 8°, 208 sagittally oriented slices, voxel size = 0.8 x 0.8 x 0.8 millimeter^3^. In addition, the monkeys underwent two baseline PET scans (Siemens MicroPET Focus 220 scanner) of three different radiotracers: [^11^C]dihydrotetrabenazine (DTBZ) which reflects VMAT2, 2beta-[^11^C]carbomethoxy-3beta-(4-fluorophenyl)tropane (CFT) which reflects DAT and [^18^F]-fluorodopa (FD) which primarily reflects decarboxylase and storage following published procedures [8,11,12]. DTBZ 168 to 386 MBq, CFT 259 to 396 MBq, or FD 112 to 422 MBq was administrated by bolus intravenous injection through the saphenous vein into each subject. PET scanning began at the start of tracer injection and continued 60 minutes (3 x 1 minute, 4 x 2 minutes, 3 x 3 minutes, and 8 x 5 minutes) for DTBZ, 115 minutes (3 x 1 minute, 4 x 2 minutes, 3 x 3 minutes, and 19 x 5 minutes) for CFT, and 95 minutes (3 x 1 minute, 4 x 2 minutes, 3 x 3 minutes, and 15 x 5 minutes) for FD. Once the primates had become proficient in their behavioral exercises and had baselines scans, a variable dose of MPTP (Sigma, St. Louis, MO) ranging from 0 (normal saline without MPTP) to 0.31 mg per kilogram body weight was infused via the right internal carotid artery [8,10]. After MPTP, the animals continued behavioral exercises for 8 weeks. During the final week (prior to euthanasia), one more structural MRI and at least two more PET scans of each tracer were performed. Following the final PET scan, primates were euthanized with an overdose of pentobarbital (100 mg/kg, i.v.) (Butler Schein Animal Health, Dublin, OH). After euthanasia, the brain was removed within 10 minutes and processed as previously reported to obtain stereologic counts of nigral dopaminergic neurons [9]. No dopaminergic drugs were administered at any time and the primates were able to take care of themselves throughout the study.

An 8-week time period was established because previous studies have shown MPTP-treated monkeys reached maximal parkinsonism and stabilized by 3 weeks after MPTP until euthanasia [8,10].

### Apathy and Motor Ratings

Although human apathy is usually defined as a loss of motivation or goal-directed behavior, the scale used in this primate study noted apathetic behavior as a lack of *willingness* to perform trained exercises [13]. Observers blinded to MPTP dose rated each video for apathetic behavior in each of the three exercises using a scale developed for primate studies [7]. The three exercises included hallway walking, circle walking, and left and right arm reaching. Baseline behavior was defined by the average of the five sessions just before MPTP while post-MPTP apathetic behavior was defined by the average of the five sessions just prior to euthanasia eight weeks after MPTP. This second set of five sessions coincided with the final set of PET scans. A Post/Pre ratio of apathetic behavior was then calculated for each primate. It is important to note that the ratings focus on the prolonged and sometimes active refusal to attempt the task rather than the performance of the movement itself. In this way, apathetic behavior could be better distinguished from motor deficits.

The sessions also were rated for parkinsonian motor symptoms. The same observer blinded to the MPTP dose rated each video using a scale previously developed and validated for non-human primate studies [8,10,14]. Parkinsonism scores ranged from 0 to 18 per side. The final motor score at 2 months after MPTP (just prior to euthanasia) was used for analysis. These motor and apathy behavioral data have been published previously [7].

### PET Analysis

**Fig 1:**
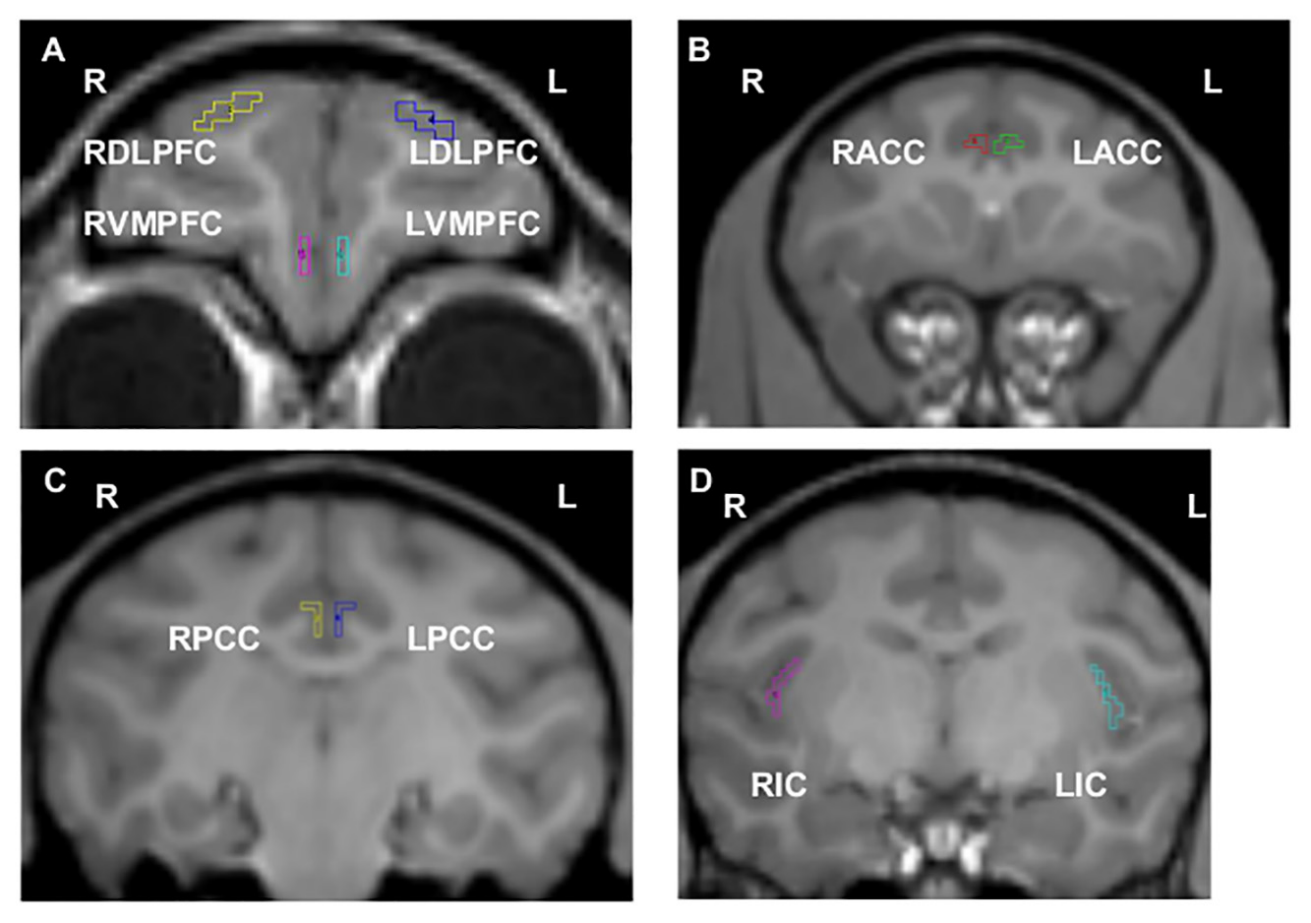
Regions of interest analyzed in brain are drawn on T1-weighted MR images. Region of interest (ROI) extended two additional coronal slices in the anterior and two in the posterior directions. ROIs are shown in right (R) and left (L) hemispheres: (A) dorsal lateral prefrontal cortex (RDLPFC, LDLPFC), and ventromedial prefrontal cortex (RVMPFC, LVMPFC), (B) anterior cingulate cortex (RACC, LACC), (C) posterior cingulate cortex (RPCC, LPCC) and (D) insular cortex (RIC, LIC).

**Fig 2:**
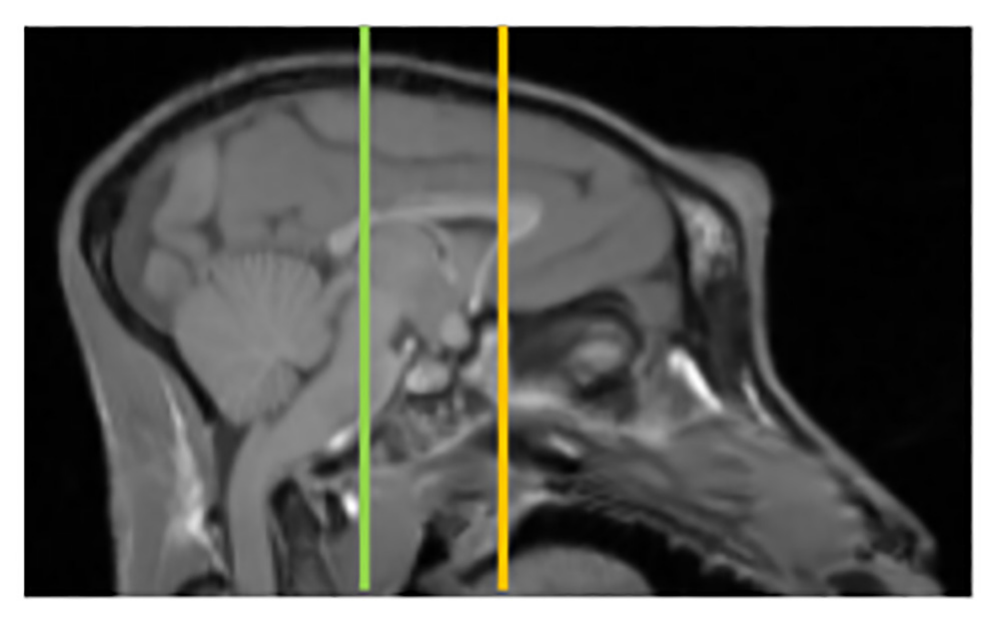
An example of how anterior cingulate cortex and posterior cingulate cortex were distinguished for tracing. A sagittal slice of a T1 weighted MR image is shown with vertical lines for the posterior commissure (green vertical line) and the most anterior part of the genu of the corpus callosum (yellow vertical line). The coronal slices used for the anterior cingulate cortex (ACC) were the five centered around the coronal slice containing the most anterior part of the genu of the corpus callosum. The coronal slices used for the posterior cingulate cortex (PCC) were the five centered around the coronal slice containing the posterior commissure.

Images were processed as previously described [7,8,11]. An investigator blinded to MPTP dose and behavioral ratings manually traced regions of interest (ROIs) on the MP-RAGE for each animal. These ROIs include DLPFC, VMPFC, ACC, PCC, IC, and occipital cortex (as a reference region) (Fig 1). The MP-RAGE image for each animal was co-registered to the baseline DTBZ scan for that animal using a voxel-based vector gradient co-registration technique [15]. All other PET scans were subsequently co-registered to this initial DTBZ scan using the same vector gradient transformation. In this study, however, the MP-RAGE was spatially normalized along the interhemispheric fissure to make the anterior-posterior axis of the brain parallel with the anterior-posterior commissure (AC-PC) line [16]. This new part of the analysis provided a substantial improvement in co-registration that permitted identification of ACC, PCC, and IC. These ROIs were drawn on this spatially normalized MP-RAGE but then mapped back to native space prior to analysis. DLPFC, VMPFC, and occipital cortex were drawn directly on the native space MRP-RAGE images. Five coronal sections were used for each ROI. The left and right DLPFC and VMPFC were based on Areas 9 and 10, respectively [17] [16]. The ACC was identified on the coronal slice that contained the most anterior part of the genu of the corpus callosum and included two coronal slices anterior and two coronal slices posterior to this central slice (see Fig 2 [16]). The PCC was identified on the coronal slice that contained the posterior commissure and included the two slices directly anterior and posterior to this central slice (see Fig 2 [18]). Finally, the five coronal slices of the IC ROI were determined by first identifying the most rostral and caudal slices of the IC [19]. After that was determined, the middle coronal slice between these two extremes was taken as the center and the two slices directly anterior and posterior to this center were included to create the IC ROI. The reference region for the PET analyses was a pair of hemi-cylinders on either side of the interhemispheric fissure in the occipital cortex. The mean VOI volumes (cm^3^) were 0.04 (DLPFC), 0.02 (VMPFC), 0.04 (ACC), 0.05 (PCC), 0.12 (IC) and 1.3 (occipital cortex). The ROIs defined in MP-RAGE space then were transformed into the PET space of the initial DTBZ scan using the transformation matrix calculated with the vector gradient co-registration. PET data were extracted for each ROI for each radiotracer. PET images for each radioligand and ROI from one representative monkey after MPTP infusion were shown in S1 Fig.

Non-displaceable binding potential (*BP*_ND_) values were calculated for CFT and DTBZ scans, and influx constants (*K*_occ_) were determined for FD scans following calculations described previously [20–23] with the occipital reference region’s uptake as the input function. An injected/control (affected right hemisphere vs. unaffected left hemisphere) ratio for *BP*_ND_ or *K*_occ_ then was calculated for each scan. The average ratio of the two pre-MPTP scans was calculated as the baseline tracer uptake and the average ratio of the two post-MPTP scans was calculated as the post-MPTP tracer uptake.

### TH Immunohistology and stereology

Following euthanasia, TH immunostaining identified dopaminergic cells for subsequent stereological cell counting following published procedures [24,25]. Unbiased stereological counts of nigrostriatal neurons stained with TH were performed on the SN and VTA for both hemispheres, and the data from these counts have been published [7].

### Statistical Analysis

Analyses were performed and plots were generated using IBM PASW Statistics software version 22.0 (IBM, Armonk, New York). The primary dependent measures were apathetic behavior scores expressed as post/pre-MPTP ratios, parkinsonian motor scores at 2 months post-MPTP, and PET measures expressed as post-MPTP injected/control hemisphere ratios. Outliers were identified from data that was 1.5 times the interquartile range, an assumption that permits study of data that adequately reflects the average in our population of interest. These outliers might arise from the inherent variability of the radiotracer uptake measures, particularly for relatively small cortical ROIs in which radioactivity counts are relatively low. To evaluate the effect of MPTP on these 5 cortical ROIs, paired t-tests and non-parametric Wilcoxon Signed Rank tests compared post-MPTP injected vs. control hemisphere uptakes. Additionally, one-way repeated measures ANOVA (rmANOVA) analyses (or the non-parametrically equivalent Friedman test) and post-hoc tests employing Bonferonni corrections for multiple comparisons were conducted to identify differential uptake of the radiotracers across the ROIs. To identify relationships between apathetic and motor behavior to the PET data, non-parametric Spearman correlations were performed between post-MPTP injected/control *BP*_ND_ or *K*_occ_ ratios and apathetic behavior or final motor score ratios. Non-parametric correlations were required because the apathy and motor measures are rank-ordered ratings. Finally, forward step-wise regression analyses were used to identify which dopaminergic pathways (SN or VTA mediated), if any, contributed to correlations between the cortical ROIs and apathetic behavior or motor ratings. Two-tailed P values less than 0.05 were considered statistically significant.

## Results

All 16 monkeys completed the study and demonstrated a wide range of motor impairment and apathetic behaviors. Parkinsonian ratings and apathy scores increased after MPTP. The parkinsonian motor ratings stabilized for all monkeys by 3 to 4 weeks post-MPTP, and apathy scores remained relatively stable over the 8 weeks post-MPTP. The details of species, age, weight, MPTP dose, parkinsonian motor score, baseline and post-MPTP apathy score for each animal are provided in S1 Table. Behavioral and biochemical measures have been reported in separate papers [7,10]. One animal’s PET data could not be analyzed for the ACC, PCC, and IC because MRI-to-PET co-registration was excessively noisy. All the data are presented for each of the analyses (n_max_ = 16 for the DLPFC and VMPFC, and n_max_ = 15 for the ACC, PCC, and IC).

### MPTP-induced changes in specific radiotracer uptake in cortical regions

Reductions in cortical uptake occurred ipsilateral to the side of MPTP injection in the VMPFC (CFT *BP*_ND_, p < 0.005), PCC (DTBZ *BP*_ND_, p < 0.05), and IC (CFT *BP*_ND_, p < 0.005; DTBZ *BP*_ND_, p < 0.05; FD *K*_occ_, p < 0.05). Radiotracer uptake also differed across the 5 cortical regions post-MPTP. CFT *BP*_ND_ and DTBZ *BP*_ND_ differed across the 5 ROIs (Friedman test: χ^2^(4) = 17.32, χ^2^(4) = 17.43, p < 0.005 for CFT *BP*_ND_ and DTBZ *BP*_ND_, respectively); CFT and DTBZ uptake in the IC was significantly reduced compared to the other 4 regions (p < 0.05) revealed by post hoc Wilcoxon signed rank tests with Bonferroni correction. S2 and S3 Tables list specific radiotracer uptake in the right and left hemispheres of pre- and post-MPTP, respectively.

### Correlations between regional radiotracer specific uptake and apathy ratings

**Fig 3:**
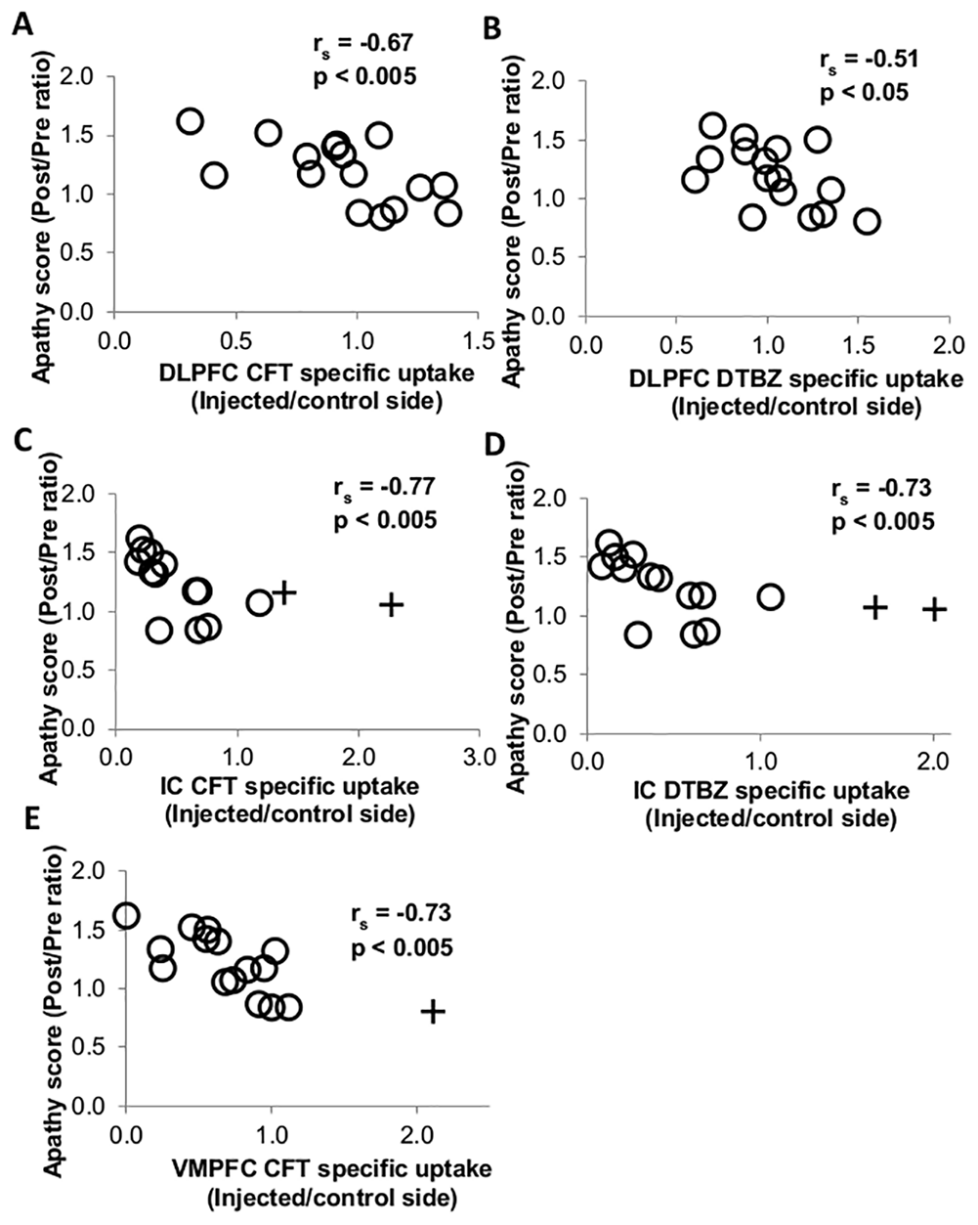
Relationship between apathy score and specific uptake of different radiotracers in different cortical regions. (A) CFT specific uptake in dorsal lateral prefrontal cortex (DLPFC), (B) DTBZ specific uptake in DLPFC, (C) CFT specific uptake in insular cortex (IC), (D) DTBZ specific uptake in IC, and (E) CFT specific uptake in ventromedial prefrontal cortex (VMPFC). + indicates outlier. Note r_s_ and *p* in Fig 3 are from data including outliers, if applicable. After excluding outliers, apathy score still significantly correlated with specific uptake of CFT in IC (r_s_ = -0.80, p < 0.005), DTBZ in IC (r_s_ = -0.76, p < 0.005), and CFT in VMPFC (r_s_ = -0.67, p < 0.05).

Radiotracer uptake in DLPFC, IC and VMPFC negatively correlated with apathetic behaviors (Fig 3A–3E) whereas uptake in ACC and PCC did not (S2A–S2F Fig). In the DLPFC, CFT *BP*_ND_ (r_s_ = -0.67, p < 0.005, n = 16) (Fig 3A) and DTBZ *BP*_ND_ (r_s_ = -0.51, p < 0.05, n = 16) (Fig 3B) negatively correlated with apathy scores. Similarly, in the IC, CFT *BP*_ND_ (r_s_ = -0.80, p < 0.005, n = 15) (Fig 3C) and DTBZ *BP*_ND_ (r_s_ = -0.76, p < 0.005, n = 15) (Fig 3D) negatively correlated with apathy ratings. In the VMPFC, CFT *BP*_ND_ (r_s_ = -0.73, p < 0.005, n = 15) negatively correlated with apathy ratings (Fig 3E), but DTBZ *BP*_ND_ did not (S2G Fig). In contrast, FD *K*_occ_ in DLPFC, IC, and VMPFC did not correlate with apathy scores (S2H–S2J Fig).

### Correlations between regional radiotracer specific uptake and parkinsonian ratings

**Fig 4:**
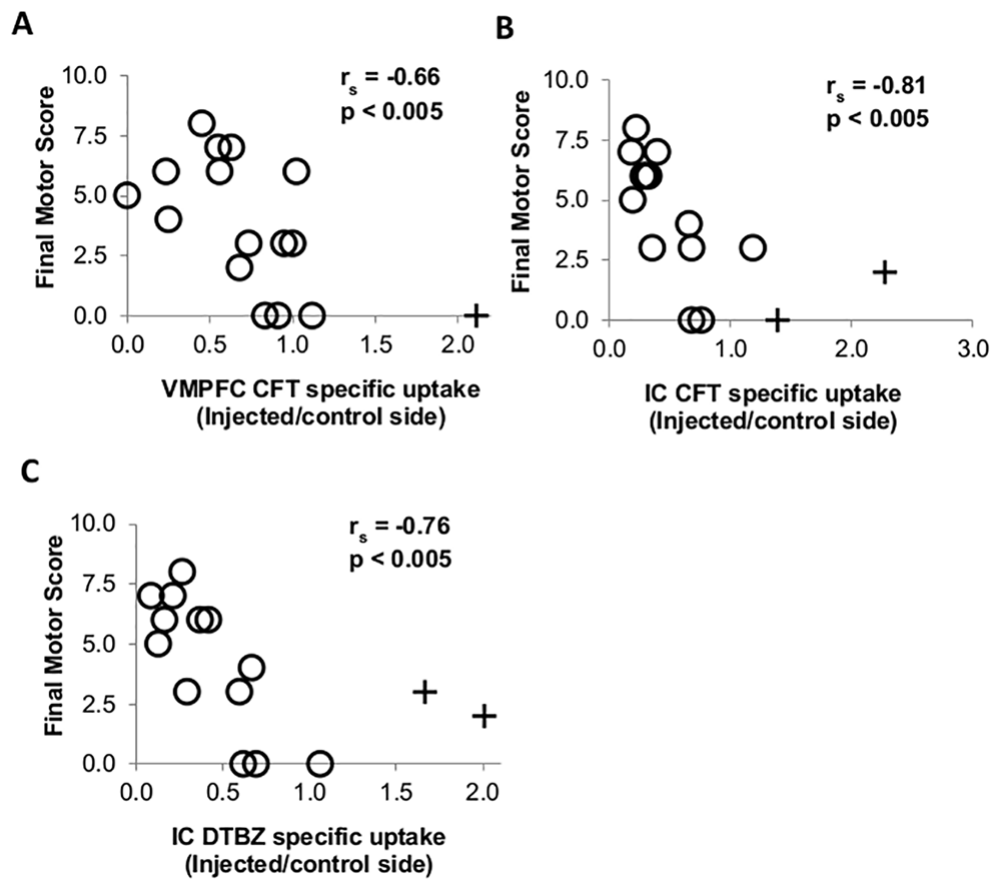
Relationship between motor score and specific uptake of different radiotracers in different cortical regions. (A) CFT specific uptake in ventromedial prefrontal cortex (VMPFC), (B) CFT specific uptake in insular cortex (IC), and (C) DTBZ specific uptake in IC. + indicates outlier. Note r_s_ and *p* in Fig 4 are from data including outliers, if applicable. After excluding outliers, motor score still significantly correlated with specific uptake of CFT in VMPFC (r_s_ = -0.60, p < 0.05), CFT in IC (r_s_ = -0.76, p < 0.005), and DTBZ in IC (r_s_ = -0.75, p < 0.005).

In VMPFC, CFT *BP*_ND_ negatively correlated with parkinsonian ratings (r_s_ = -0.66, p < 0.005, n = 16) (Fig 4A) but DTBZ BP_ND_ did not (S3A Fig). In IC, CFT *BP*_ND_ (r_s_ = -0.81, p < 0.005, n = 15) (Fig 4B) and DTBZ *BP*_ND_ (r_s_ = -0.76, p < 0.005, n = 15) (Fig 4C) negatively correlated with parkinsonian ratings. In contrast, FD *K*_occ_ in either VMPFC or IC did not correlate with motor ratings (S3B and S3C Fig); parkinsonian ratings did not correlate with any of the three radiotracers in the ACC, PCC, and DLPFC (S3D–S3L Fig).

### Using subcortical dopaminergic pathways to predict cortical uptake

To determine whether the changes in uptake of the presynaptic dopaminergic radiotracers in these cortical regions related more to either SN or VTA mediated dopaminergic pathways, we factored SN and VTA TH-stained unbiased stereologic cell counts into forward step-wise regression analyses. An inclusion level of p < 0.05 was set. When IC CFT *BP*_ND_ values were set as the dependent variable and SN and VTA cell count ratios were placed as independent variables, VTA cell counts were a significant predictor of IC CFT *BP*_ND_ values (adjusted R^2^ = 0.43, F(1, 10) = 9.23, p < 0.05) while SN ratios were excluded from the model (p = 0.44). On the other hand, when DLPFC or VMPFC CFT *BP*_ND_ values were set as the dependent variable with SN and VTA cell count ratios as independent variables, neither of the two independent variables met the inclusion level and no significant model resulted.

## Discussion

This study found that apathetic behavior changes correlate with impairments in presynaptic dopaminergic markers in cortical regions in monkeys with a wide spectrum of severity of nigral dopaminergic neuronal injury induced by unilateral MPTP infusion. For the same set of monkeys, our group already demonstrated that integrity of the VTA-NAcc pathway more strongly predicts apathetic behaviors than the SN-mediated pathway. In the present study, we have substantially extended these findings with two key findings. First, increased apathy scores correlated with PET measured changes in the VTA dopaminergic cortical projections to DLPFC, VMPFC, and IC. Second, compared to nigrostriatal dysfunction, decrease in VTA dopaminergic neurons better predicted reduced DAT in the IC.

### Apathy rating correlates with dopaminergic dysfunction in the VMPFC, DLPFC, and IC

We found that apathy ratings correlated with changes in DLPFC, VMPFC and IC as reflected by PET measured changes in uptake of VMAT2 and DAT radiotracers in these regions. These results support the notion that dysfunction of the prefrontal cortex-basal ganglia circuits plays a role in the pathology of apathy [1]. Previous functional imaging studies have shown hypometabolism in the medial frontal cortex and in the DLPFC in non-demented PD participants, as compared to healthy controls when performing a motor task [3,5,26]. Damage to human VMPFC leads to the loss of modulatory control of voluntary actions [27]. More specifically, apathy has been associated with localized IC infarction [28] and volume reduction of the IC [19]. Our present study found that apathetic behavior severity negatively correlated with dopaminergic dysfunction in the IC, as revealed by decreased DAT and VMAT2 specific binding. In contrast, we found no correlation between apathetic behavior severity and dopaminergic pathway dysfunction in the ACC and PCC whereas previous studies of apathy in humans found a relationship with dysfunction of the ACC and PCC. For example, people with apathy associated with Alzheimer disease have ACC hypoperfusion [29,30] as measured by single photon emission computed tomography (SPECT), while people with PD may have subthalamic deep brain stimulation-induced apathy that corresponds to stimulation-induced decreases in glucose metabolism in the PCC [31]. These discrepancies between our MPTP-induced animal model and the human studies in Alzheimer and PD with deep brain stimulation may reflect the manipulations done in the participants (like deep brain stimulation) and the difference between Alzheimer and PD pathology. Clearly, Alzheimer disease has a different pathology from MPTP-induced dopaminergic deficits. Perhaps, more importantly, people with PD have more than dopaminergic deficits since synucleinopathy extends beyond dopaminergic pathways [32]. Nevertheless, correlations between apathetic behaviors and dopaminergic dysfunction in multiple cortical regions suggest that neuronal networks rather than single cortical regions are important for preservation of complex goal-direct behaviors. Therefore, our data provide further evidence that reduced dopaminergic innervation from VTA to the DLPFC, VMPFC, and IC plays a role in apathetic behaviors.

### Degeneration of the VTA-IC dopaminergic system

We found that loss of dopaminergic neurons in VTA predicated DAT loss in the IC, whereas SN dopaminergic neuronal loss did not. The IC connects with VTA anatomically [33] and functionally [34]. Anatomically, the IC has extensive reciprocal connections with other cortical regions [35], and the ICreceives its greatest innervation from VTA [36]. The IC is multifunctional and is associated with viscerosensory, visceromotor, vestibular, somatosensory, and somatomotor control functions [37]. For example, two meta-analyses of functional imaging data indicated that motor tasks activate the IC [38,39]. Moreover, the IC, among many other cortical areas, is involved in a vestibular cortical network [40] and plays a critical role in vestibular functions [41]; patients with unilateral lesions in the IC showed body lateropulsion [42]. VTA-IC dopaminergic dysfunction might directly contribute to dysfunction of the IC. Furthermore, dopamine depletion in the IC exceeds depletion in cortical regions in postmortem studies of PD patients [43]. Therefore, the progressive VTA-IC dopaminergic dysfunction demonstrated here may contribute to some of apathetic behaviors in MPTP-treated monkeys.

Our group has previously reported that dysfunction in VTA-NAcc pathway contributes more to the manifestation of apathy than nigrostriatal pathway deficits [7]. In the current study, we extended these findings to including dysfunction of VTA-IC pathways. These findings support a key role for the VTA involving both subcortical and now cortical regions in the pathophysiology of apathy.

### Limitations of the Study

Some limitations should be noted. First, the present study used an MPTP-lesioned monkey model that does not necessarily mimic the pathology seen in PD patients, although this model provides a model of dopaminergic loss in the SNpc and VTA. Alternatively, chronic low-dose intoxication with MPTP in primate might produce a different pattern of nigrostriatal denervation from acute high-dose intoxication. Chronic MPTP administration may induce slightly greater dopamine depletion in the putamen than the caudate, and increase dopamine turnover, whereas acute MPTP treatment does not affect dopamine turnover [44,45]. Second, although the apathetic behavior rating scale was validated, this scale used in monkeys only assesses apathy by observable behaviors and cannot include reports of thoughts or moods, which are critical components of apathy in humans. Consequently, we could not measure deficits in internal thoughts which play a role in goal-directed behavior. Third, MPTP can affect other neurotransmitter systems including norepinephrine (NE) and serotonin (5HT) system in the frontal cortex and other extrastriatal regions [46–48]. Furthermore, serotonergic and cholinergic systems in other cortical and subcortical regions likely play a role in apathetic behaviors [49,50]. Our *in vivo* PET measures only interrogated alterations in the dopaminergic system in several cortical regions. Further studies will be needed to investigate the role of these other transmitter systems.

In conclusion, we found that apathy ratings correlated with changes in the VTA dopaminergic projections to DLPFC, VMPFC and IC. Additionally, we demonstrated that dysfunction of the VTA-IC pathway contributes to apathetic behaviors and motor impairments. Our study substantially extends previous observations relating apathy to dysfunction of distinct cortical regions. Additional studies are needed to determine the role of other brain regions and other neurotransmitter systems in the pathophysiology of apathy.

## Supporting Information

S1 Fig Representative PET images of 3 radiotracers in cortical regions for one monkey after MPTP infusion. PET images with region of interests (ROIs) overlaid for post-MPTP scans from one monkey are shown for CFT, DTBZ, and CFT in top, middle and bottom row, respectively. The ROIs from left to right side are anterior cingulate cortex (ACC), posterior cingulate cortex (PCC), insular cortex (IC), dorsal lateral prefrontal cortex (DLPFC), and ventromedial prefrontal cortex (VMPFC). Note the left and right VMPFC seemed overlapped due to limited resolution of image software, but they were distinctly separated on MP-RAGE and subsequent radiotracer uptake analyses.
(TIF)

S2 Fig Relationship between apathy score and specific uptake of different radiotracers in different cortical regions. (A) CFT specific uptake in anterior cingulate cortex (ACC), (B) DTBZ specific uptake in ACC, (C) FD specific uptake in ACC, (D) CFT specific uptake in posterior cingulate cortex (PCC), (E) DTBZ specific uptake in PCC, (F) FD specific uptake in PCC, (G) DTBZ specific uptake in ventromedial prefrontal cortex (VMPFC), (H) FD specific uptake in dorsal lateral prefrontal cortex (DLPFC), (I) FD specific uptake in insular cortex (IC), and (J) FD specific uptake in VMPFC. Each point represents the post/pre-MPTP apathy score and post-MPTP injected/control side measures for an individual subject, and each line is the linear fit of the data. + indicates outlier. Note r_s_ and p in this figure are from data including outliers, if applicable.
(TIF)

S3 Fig Relationship between motor score and specific uptake of different radiotracers in different cortical regions. (A) DTBZ specific uptake in ventromedial prefrontal cortex (VMPFC), (B) FD specific uptake in VMPFC, (C) FD specific uptake in insular cortex (IC), (D) CFT specific uptake in anterior cingulate cortex (ACC), (E) DTBZ specific uptake in ACC, (F) FD specific uptake in ACC, (G) CFT specific uptake in PCC, (H) DTBZ specific uptake in posterior cingulate cortex (PCC), (I) FD specific uptake in PCC, (J) DTBZ specific uptake in dorsal lateral prefrontal cortex (DLPFC), (K) CFT specific uptake in DLPFC, and (L) FD specific uptake in DLPFC. Each point represents the post/pre-MPTP apathy score and post-MPTP injected/control side measures for an individual subject, and each line is the linear fit of the data. + indicates outlier. Note r_s_ and p in this figure are from data including outliers, if applicable.
(TIF)

S1 Table Summary of each monkey's species, age at MPTP injection, weight, MPTP dose, parkinsonism score, and apathy score. Monkey numbers with superscript a indicate *Macaca nemestrina*, all others without a label are *Macaca fascicularis*. Weight is given in kg at MPTP injection, MPTP dose is given in mg/kg body weight, and motor score is the final score at 2 months post-MPTP.
(PDF)

S2 Table Mean measurement of specific uptake of 3 radiotracers for different cortical regions pre-MPTP infusion. Data in parentheses represent values after excluding outliers. SD: standard deviation; *BP*_ND_: non-displaceable binding potential; *K*_occ_: influx constants; DLPFC: dorsal lateral prefrontal cortex; VMPFC: ventromedial prefrontal cortex; ACC: anterior cingulate cortex; PCC: posterior cingulate cortex; IC: insular cortex.
(PDF)

S3 Table Mean measurement of specific uptake of 3 radiotracers for different cortical regions after MPTP infusion. Note that post-MPTP measures reflect changes following a wide range of MPTP doses, not a uniform dose. Significant differences are indicated by * (*p* < 0.05) or ** (*p* < 0.005) as determined by Wilcoxon signed-rank tests or paired t-tests. Data in parentheses represent values after excluding outliers. SD: standard deviation; *BP*_ND_: non-displaceable binding potential; *K*_occ_: influx constants; DLPFC: dorsal lateral prefrontal cortex; VMPFC: ventromedial prefrontal cortex; ACC: anterior cingulate cortex; PCC: posterior cingulate cortex; IC: insular cortex.
(PDF)
