# Supplementary figures and images for: Neuroimaging Analysis of the Dopamine Basis for Apathetic Behaviors in an MPTP-Lesioned Primate Model
Source: PLoS One. 2015 Jul 2;10(7):e0132064. doi: 10.1371/journal.pone.0132064 (PMC4489892; doi:10.1371/journal.pone.0132064)

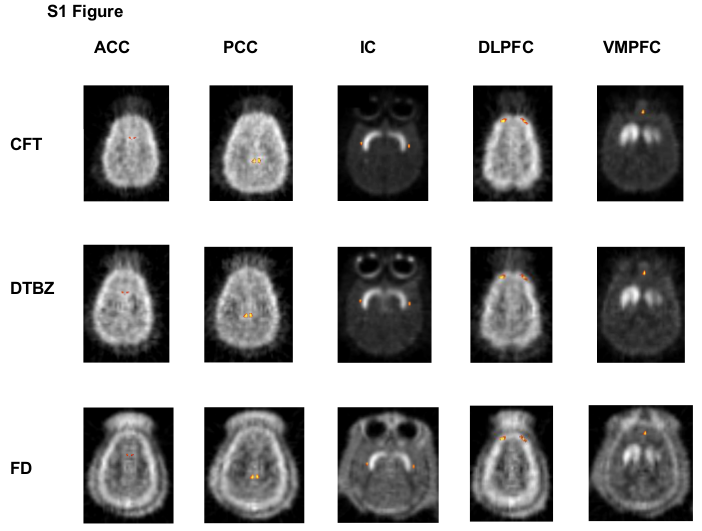

Supplement: S1 Fig — PET images with region of interests (ROIs) overlaid for post-MPTP scans from one monkey are shown for CFT, DTBZ, and CFT in top, middle and bottom row, respectively. The ROIs from left to right side are anterior cingulate cortex (ACC), posterior cingulate cortex (PCC), insular cortex (IC), dorsal lateral prefrontal cortex (DLPFC), and ventromedial prefrontal cortex (VMPFC). Note the left and right VMPFC seemed overlapped due to limited resolution of image software, but they were distinctly separated on MP-RAGE and subsequent radiotracer uptake analyses. (TIF) [file pone.0132064.s001.tif]

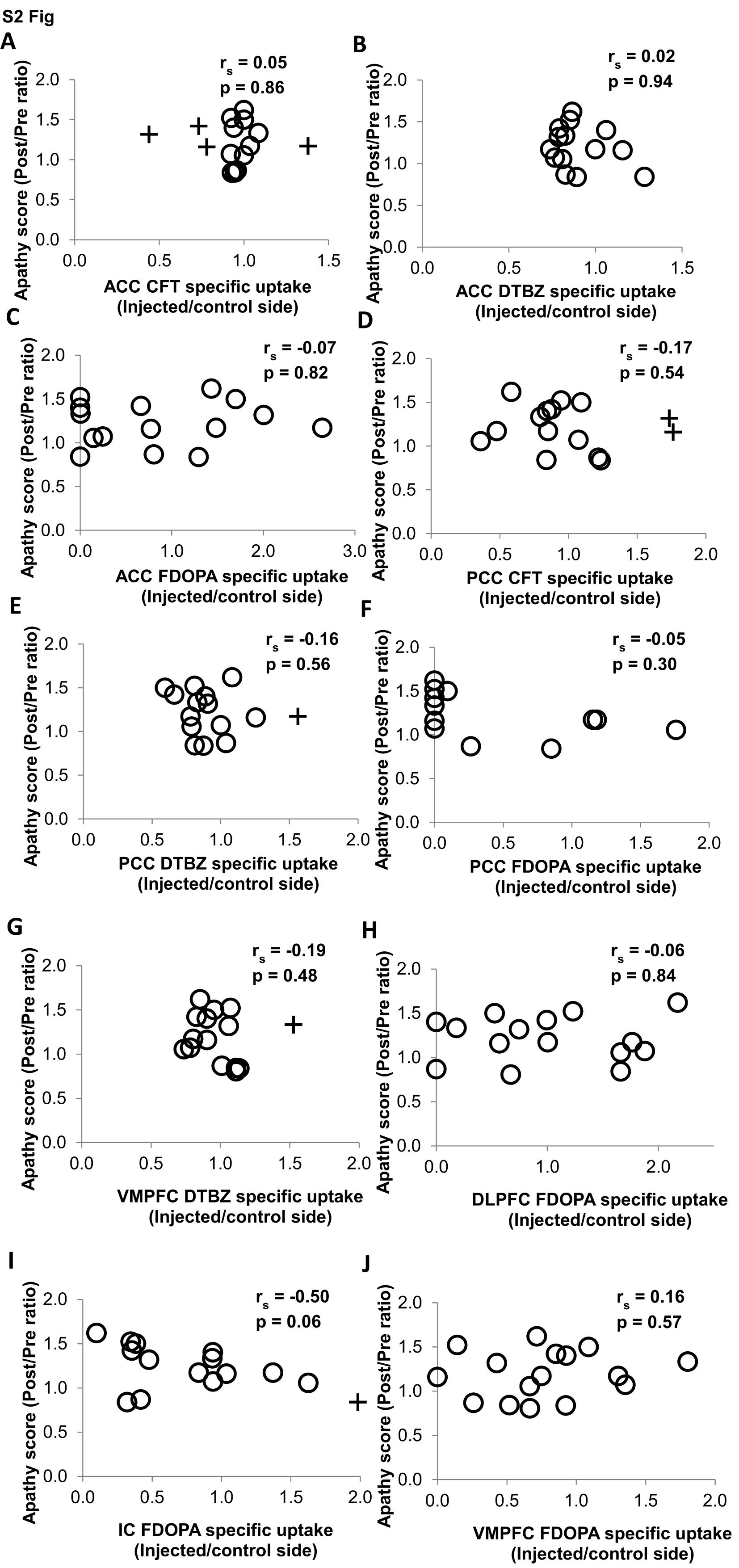

Supplement: S2 Fig — (A) CFT specific uptake in anterior cingulate cortex (ACC), (B) DTBZ specific uptake in ACC, (C) FD specific uptake in ACC, (D) CFT specific uptake in posterior cingulate cortex (PCC), (E) DTBZ specific uptake in PCC, (F) FD specific uptake in PCC, (G) DTBZ specific uptake in ventromedial prefrontal cortex (VMPFC), (H) FD specific uptake in dorsal lateral prefrontal cortex (DLPFC), (I) FD specific uptake in insular cortex (IC), and (J) FD specific uptake in VMPFC. Each point represents the post/pre-MPTP apathy score and post-MPTP injected/control side measures for an individual subject, and each line is the linear fit of the data. + indicates outlier. Note rs and p in this figure are from data including outliers, if applicable. (TIF) [file pone.0132064.s002.tif]

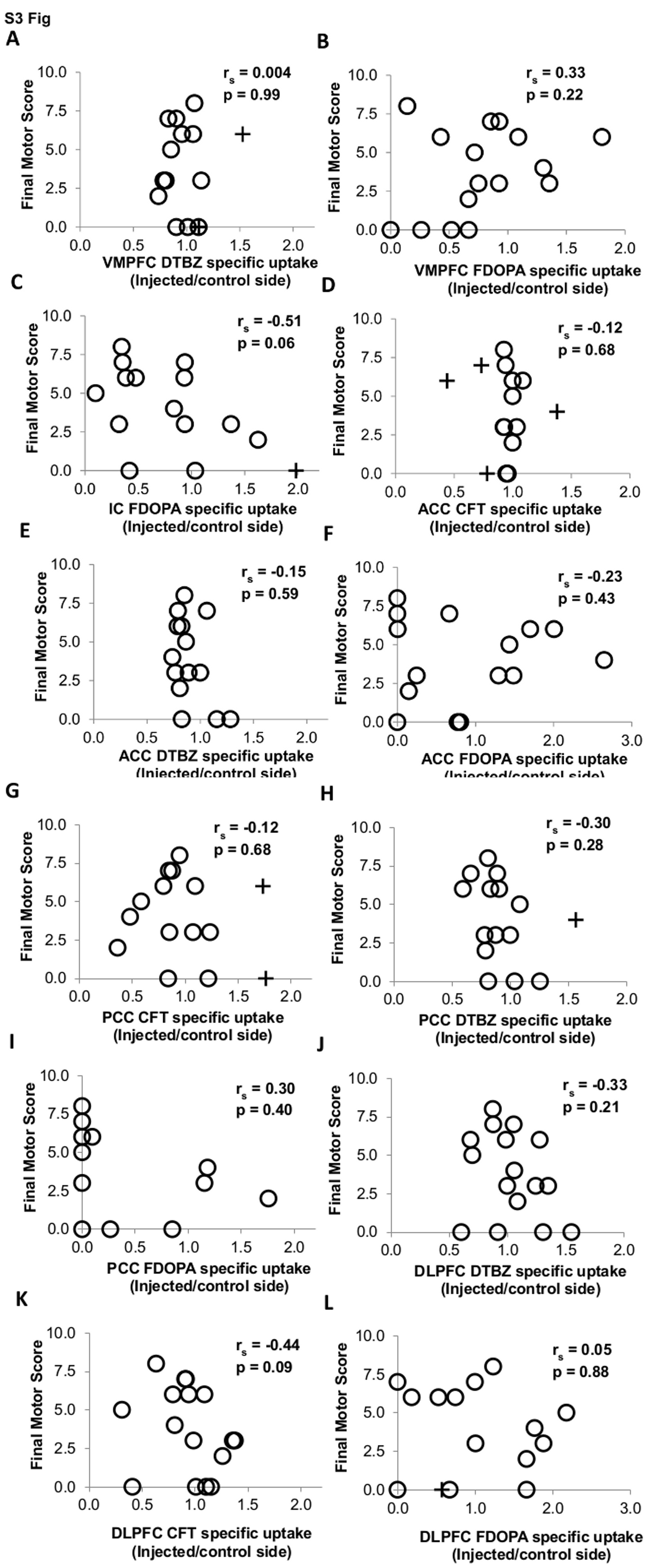

Supplement: S3 Fig — (A) DTBZ specific uptake in ventromedial prefrontal cortex (VMPFC), (B) FD specific uptake in VMPFC, (C) FD specific uptake in insular cortex (IC), (D) CFT specific uptake in anterior cingulate cortex (ACC), (E) DTBZ specific uptake in ACC, (F) FD specific uptake in ACC, (G) CFT specific uptake in PCC, (H) DTBZ specific uptake in posterior cingulate cortex (PCC), (I) FD specific uptake in PCC, (J) DTBZ specific uptake in dorsal lateral prefrontal cortex (DLPFC), (K) CFT specific uptake in DLPFC, and (L) FD specific uptake in DLPFC. Each point represents the post/pre-MPTP apathy score and post-MPTP injected/control side measures for an individual subject, and each line is the linear fit of the data. + indicates outlier. Note rs and p in this figure are from data including outliers, if applicable. (TIF) [file pone.0132064.s003.tif]
