# Supplementary material for: Neuroimaging Analysis of the Dopamine Basis for Apathetic Behaviors in an MPTP-Lesioned Primate Model
Source: PLoS One. 2015 Jul 2;10(7):e0132064. doi: 10.1371/journal.pone.0132064 (PMC4489892; doi:10.1371/journal.pone.0132064)
Supplement: S1 Table — Monkey numbers with superscript a indicate Macaca nemestrina, all others without a label are Macaca fascicularis. Weight is given in kg at MPTP injection, MPTP dose is given in mg/kg body weight, and motor score is the final score at 2 months post-MPTP. (PDF) [file pone.0132064.s004.pdf]

S1 Table

Monkey number	Age	Weight	MPTP dose	Motor score	Apathy	
					Pre-MPTP	Post-MPTP
1	5.8	6.6	0	0	10	8.4
2	5.6	7.0	0	0	9	7.8
3	4.9	4.9	0.07	0	9.2	7.4
4	5.6	7.0	0.07	3	10.4	11.1
5[a]	3.5	5.6	0.08	3	7	8.2
6[a]	3.7	5.1	0.12	3	7.4	6.2
7	5.3	5.6	0.13	2	6.8	7.2
8	4.5	5.7	0.13	0	8.8	10.2
9	6.3	8.2	0.13	6	8.4	11.2
10	6.3	8.9	0.14	8	9.6	14.6
11	6.4	7.5	0.14	4	8.2	9.6
12	6.4	6.9	0.14	5	8.4	13.6
13	5.1	8.9	0.19	6	10.4	13.7
14[a]	4.1	7.8	0.2	7	7	9.8
15	5.5	7.5	0.24	6	6.67	10
16	5.6	7.3	0.31	7	7.6	10.8
