# Supplementary material for: Neuroimaging Analysis of the Dopamine Basis for Apathetic Behaviors in an MPTP-Lesioned Primate Model
Source: PLoS One. 2015 Jul 2;10(7):e0132064. doi: 10.1371/journal.pone.0132064 (PMC4489892; doi:10.1371/journal.pone.0132064)
Supplement: S2 Table — Data in parentheses represent values after excluding outliers. SD: standard deviation; BP ND: non-displaceable binding potential; K occ: influx constants; DLPFC: dorsal lateral prefrontal cortex; VMPFC: ventromedial prefrontal cortex; ACC: anterior cingulate cortex; PCC: posterior cingulate cortex; IC: insular cortex. (PDF) [file pone.0132064.s005.pdf]

S2 Table

Measure	Right hemisphere mean ± SD	Left hemisphere mean ± SD
DLPFC CFT BP_{ND}	0.46±0.18	0.46±0.17
DLPFC DTBZ BP_{ND}	0.48±0.21 (0.45±0.16)	0.49±0.24
DLPFC FD K_{occ}	0.004±0.002 (0.003±0.002)	0.003±0.001
VMPFC CFT BP_{ND}	0.34±0.13 (0.38±0.08)	0.41±0.20
VMPFC DTBZ BP_{ND}	0.32±0.14	0.35±0.16
VMPFC FD K_{occ}	0.002±0.001	0.003±0.001
ACC CFT BP_{ND}	0.34±0.14	0.35±0.13
ACC DTBZ BP_{ND}	0.42±0.17	0.43±0.16
ACC FD K_{occ}	0.002±0.001	0.002±0.001
PCC CFT BP_{ND}	0.21±0.09 (0.21±0.03)	0.19±0.08
PCC DTBZ BP_{ND}	0.23±0.10	0.24±0.09
PCC FD K_{occ}	0.001±0.001	0.001±0.001
IC CFT BP_{ND}	0.69±0.30	0.70±0.20
IC DTBZ BP_{ND}	0.63±0.29	0.65±0.24
IC FD K_{occ}	0.003±0.001	0.003±0.001
