# Supplementary material for: Neuroimaging Analysis of the Dopamine Basis for Apathetic Behaviors in an MPTP-Lesioned Primate Model
Source: PLoS One. 2015 Jul 2;10(7):e0132064. doi: 10.1371/journal.pone.0132064 (PMC4489892; doi:10.1371/journal.pone.0132064)
Supplement: S3 Table — Note that post-MPTP measures reflect changes following a wide range of MPTP doses, not a uniform dose. Significant differences are indicated by * (p < 0.05) or ** (p < 0.005) as determined by Wilcoxon signed-rank tests or paired t-tests. Data in parentheses represent values after excluding outliers. SD: standard deviation; BP ND: non-displaceable binding potential; K occ: influx constants; DLPFC: dorsal lateral prefrontal cortex; VMPFC: ventromedial prefrontal cortex; ACC: anterior cingulate cortex; PCC: posterior cingulate cortex; IC: insular cortex. (PDF) [file pone.0132064.s006.pdf]

S3 Table

Measure	Right hemisphere mean ± SD	Left hemisphere mean ± SD
DLPFC CFT BP_{ND}	0.39±0.17	0.42±0.14 (0.40±0.10)
DLPFC DTBZ BP_{ND}	0.41±0.18 (0.38±0.14)	0.40±0.16 (0.37±0.12)
DLPFC FD K_{occ}	0.004±0.003 (0.003±0.002)	0.004±0.003
VMPFC CFT BP_{ND}**	0.24±0.12	0.32±0.12
VMPFC DTBZ BP_{ND}	0.31±0.12	0.32±0.13
VMPFC FD K_{occ}	0.001±0.001	0.002±0.001
ACC CFT BP_{ND}	0.31±0.11	0.33±0.11
ACC DTBZ BP_{ND}	0.39±0.12	0.41±0.16 (0.38±0.13)
ACC FD K_{occ}	0.002±0.001	0.002±0.001 (0.002±0.001)
PCC CFT BP_{ND}	0.17±0.08	0.18±0.06
PCC DTBZ BP_{ND}*	0.20±0.08 (0.18±0.06)	0.22±0.09
PCC FD K_{occ}	0.001±0.001 (0.001±0.001)	0.001±0.001 (0.001±0.001)
IC CFT BP_{ND}**	0.44±0.27	0.79±0.26 (0.75±0.21)
IC DTBZ BP_{ND}*	0.38±0.30	0.67±0.22
IC FD K_{occ}*	0.002±0.001 (0.002±0.001)	0.003±0.002 (0.003±0.001)
